# The vision and scope of the prophylactic dressing standard initiative of the European Pressure Ulcer Advisory Panel and National Pressure Injury Advisory Panel

**DOI:** 10.1111/iwj.13859

**Published:** 2022-06-14

**Authors:** David Brienza, Amit Gefen, Michael Clark, Joyce Black

**Affiliations:** ^1^ Departments of Rehabilitation Science and Technology and Bioengineering and the McGowan Institute of Regenerative Medicine University of Pittsburgh Pittsburgh Pennsylvania USA; ^2^ The Herbert J. Berman Chair in Vascular Bioengineering, Department of Biomedical Engineering, Faculty of Engineering Tel Aviv University Tel Aviv Israel; ^3^ Welsh Wound Innovation Centre Ynysmaerdy Wales UK; ^4^ School of Nursing and Midwifery Birmingham City University Birmingham UK; ^5^ Adult Health and Illness Department College of Nursing, University of Nebraska Medical Center Omaha Nebraska USA

National and international standards exist to enable testing of the properties and performance of dressings used upon wounded skin and soft tissues. However, these same dressings can and are often used upon intact skin to provide an effective component of pressure ulcer/injury prevention.^1^, ^2^, ^3^, ^4^, ^5^, ^6^ To date, there are no established and accepted standards that can be used to evaluate the prophylactic function of dressing materials.

The use of dressings for wound prevention is recommended in international clinical practice guidelines^7^ and has become the standard of care in some clinical settings. The industry has responded to the market demands stemming from these promising outcomes by promoting the use of existing wound dressings for prophylactic use. Unfortunately, two factors conspire to cause considerable problems for all parties including clinicians who want to use prophylactic dressings, for regulators and third‐party payors who need to know which products to fund in specific clinical circumstances, and also for manufacturers seeking to both improve their products and develop new products (including specific for prophylactic use) First, the clinical studies reported to date have not been designed such that they elucidate the mechanism of action of the prophylactic effects. Second, the products that have been evaluated and/or marketed for prophylactic use have substantially different characteristics related to pressure ulcer/injury prevention. For example, some provide more cushioning, while others insulate heat more than comparable products indicated for the same clinical purpose. Without knowing which characteristics affect outcomes and without having standard methods for comparing performance, none of the stakeholders have all the information needed to make proper choices for prescribing, developing, improving, and reimbursing wound dressings when they are being used to prevent pressure ulcers/injuries.

Responding to this need, the European Pressure Ulcer Advisory Panel (EPUAP) and the National Pressure Injury Advisory Panel (NPIAP) have joined forces with clinicians, manufacturers, researchers, and others to form the Prophylactic Dressing Standards Initiative (PDSI) to develop methods specifically for assessing performance of prophylactic dressings. The PDSI's intent is to eventually codify these laboratory‐based methods as international standards. The PDSI was launched in the spring of 2021 with strong support from all stakeholders. The group consists of experts from dressing manufacturers, research organisations, clinicians, and testing experts. The group has made significant progress during the first year. The PDSI participants have organised into working groups that are developing and evaluating test methodologies for individual classifications of performance including mechanical behaviour and durability, thermal performance, moisture management, and adhesiveness properties. Each working group has developed a scope of work to include identifying potential metrics, terminology, and test methods. As a guiding philosophy, PDSI has adopted a rigorous validation requirement for any new methods that might be developed by the group. For example, the working group focusing on moisture management is currently reviewing alternatives for synthetic sweat test fluids for moisture handling testing of dressings in prophylactic use. Similarly, the working group on adhesiveness reviews options for skin‐mimicking substrata, the appropriate adherence time before a peel testing, and the temperature at which peel tests should be conducted.

PDSI's activities are supported by participation fees and in‐kind contributions from members and member organisations. The targeted date for completion of initial test methods is by the spring of 2024. At that time, it is anticipated that an international standards committee will be formed within the International Organisation of Standardisation committee structure, to advance identified and developed methods as recognised international standards. The PDSI initiative provides an important example of academic, clinical, and commercial partnership to fill the current void around the technical evaluation of dressing materials used to support pressure ulcer/injury prevention. Standards exist for wound dressings concerning their characteristics related to treatment of existing wounds (EN 13726^8^). However, EN 13726 has been criticised in the literature for lack of clinical relevance.^9^ Moreover, the application of dressings for prophylaxis is not currently addressed in any existing test standard, including in the EN 13726. Accordingly, and despite there being some overlap between characteristics important to pressure ulcer/injury prevention and factors important to wound healing, the PDSI chose to limit its scope to prophylactic use of dressings only, and as related to pressure ulcers/injuries (but not other wound aetiologies), to focus energy on the current urgent need for validated methods to compare dressing products for these preventative purposes.

The multidisciplinary group will provide updates about their work at national meetings and via editorials. Please direct specific questions to the members of the guidance group, who are authors of this material.
